# Predicting Adverse Radiation Effects in Brain Tumors After Stereotactic Radiotherapy With Deep Learning and Handcrafted Radiomics

**DOI:** 10.3389/fonc.2022.920393

**Published:** 2022-07-13

**Authors:** Simon A. Keek, Manon Beuque, Sergey Primakov, Henry C. Woodruff, Avishek Chatterjee, Janita E. van Timmeren, Martin Vallières, Lizza E. L. Hendriks, Johannes Kraft, Nicolaus Andratschke, Steve E. Braunstein, Olivier Morin, Philippe Lambin

**Affiliations:** ^1^ The D-Lab, Department of Precision Medicine, GROW- School for Oncology and Reproduction, Maastricht University, Maastricht, Netherlands; ^2^ Department of Radiology and Nuclear Medicine, GROW – School for Oncology and Reproduction, Maastricht University Medical Centre+, Maastricht, Netherlands; ^3^ Department of Radiation Oncology, University Hospital of Zurich, University of Zurich, Zurich, Switzerland; ^4^ Medical Physics Unit, Department of Oncology, Faculty of Medicine, McGill University, Montréal, QC, Canada; ^5^ Department of Computer Science, Université de Sherbrooke, Sherbrooke, QC, Canada; ^6^ Department of Pulmonary Diseases, GROW – School for Oncology and Reproduction, Maastricht University Medical Centre+, Maastricht, Netherlands; ^7^ Department of Radiation Oncology, University Hospital Würzburg, Würzburg, Germany; ^8^ Department of Radiation Oncology, University of California San Francisco, San Francisco, CA, United States

**Keywords:** brain metastases (BMs), radiation necrosis (RN), deep learning - artificial neural network, radiomics, MRI, adverse radiation effects

## Abstract

**Introduction:**

There is a cumulative risk of 20–40% of developing brain metastases (BM) in solid cancers. Stereotactic radiotherapy (SRT) enables the application of high focal doses of radiation to a volume and is often used for BM treatment. However, SRT can cause adverse radiation effects (ARE), such as radiation necrosis, which sometimes cause irreversible damage to the brain. It is therefore of clinical interest to identify patients at a high risk of developing ARE. We hypothesized that models trained with radiomics features, deep learning (DL) features, and patient characteristics or their combination can predict ARE risk in patients with BM before SRT.

**Methods:**

Gadolinium-enhanced T1-weighted MRIs and characteristics from patients treated with SRT for BM were collected for a training and testing cohort (*N* = 1,404) and a validation cohort (*N* = 237) from a separate institute. From each lesion in the training set, radiomics features were extracted and used to train an extreme gradient boosting (XGBoost) model. A DL model was trained on the same cohort to make a separate prediction and to extract the last layer of features. Different models using XGBoost were built using only radiomics features, DL features, and patient characteristics or a combination of them. Evaluation was performed using the area under the curve (AUC) of the receiver operating characteristic curve on the external dataset. Predictions for individual lesions and per patient developing ARE were investigated.

**Results:**

The best-performing XGBoost model on a lesion level was trained on a combination of radiomics features and DL features (AUC of 0.71 and recall of 0.80). On a patient level, a combination of radiomics features, DL features, and patient characteristics obtained the best performance (AUC of 0.72 and recall of 0.84). The DL model achieved an AUC of 0.64 and recall of 0.85 per lesion and an AUC of 0.70 and recall of 0.60 per patient.

**Conclusion:**

Machine learning models built on radiomics features and DL features extracted from BM combined with patient characteristics show potential to predict ARE at the patient and lesion levels. These models could be used in clinical decision making, informing patients on their risk of ARE and allowing physicians to opt for different therapies.

## 1 Introduction

Brain metastases (BM) are the most common intracranial malignancies, accounting for more than 50% of all brain tumors and occurring in 10 to over 40% of patients with solid malignancies (1–3). BM occur most often in patients with lung cancer, breast cancer, and melanoma, which have a cumulative risk ranging from 20 to 40% of developing BM (4–7). BM can be treated locally by surgery or radiotherapy or with systemic anticancer therapy. Treatment depends on several factors, such as patient performance status, number and volume of metastases, presence of extracranial metastases, symptoms, and presumed efficacy of available systemic therapy [“Systemic therapy for brain metastases”, (8, 9). The radiotherapy of BM can be either stereotactic radiotherapy (SRT) or whole brain radiotherapy (WBRT), with SRT being the guideline-recommended treatment for a limited number of BM. As WBRT is associated with neurocognitive deterioration, SRT is increasingly used in multiple BM as well (10–12). SRT is delivered either in a single fraction, with stereotactic radiosurgery (SRS), or as fractionated stereotactic radiotherapy (FSRT) and results in a high dose within the target volume with a steep dose gradient to the surrounding healthy tissue (13).

Even though most of the healthy brain is spared from high doses of radiation, a major shortcoming of SRT is a chance of high toxicity in the immediate surrounding tissues, which may lead to adverse radiation effects (ARE) such as radiation necrosis (RN), subacute edema, structural changes in the white matter, and vascular lesions (14). ARE are a relatively late reaction to irradiation of healthy tissues where either reversible or irreversible injury has occurred (15). The risk of ARE after SRT and SRS is found to be similar and ranges from 5 to 10% at patient level (16–19) or approximately 3% at lesion level (15). Known predictors of ARE are tumor volume, isodose volume, and previous SRT to the same lesion (15). ARE of the tumor area and tumor progression (TP) as two different post-therapeutic events require different treatment strategies: while steroids are often indicated for the initial treatment of ARE, true progression or relapse requires repeated radiotherapy, surgery, or effective intracranial systemic therapy for tumor control. Being able to differentiate between ARE and TP is therefore of utmost clinical interest.

Unfortunately, the (neurological) symptoms of ARE and TP are usually indistinguishable. Furthermore, the appearances of ARE and TP are very difficult to discern through qualitative radiological imaging, requiring multiple successive magnetic resonance images (MRI), specialized MRI sequences such as perfusion-weighted or MR spectroscopy, and trained experts to evaluate the findings (19, 20). The clinical workflow is time- and labor-intensive, and while it is unfeasible to perform for every lesion, a definitive confirmation of the presence of ARE requires tissue acquisition (19).

SRT requires routine pretreatment MRI for accurate target volume delineation. This imaging provides a source of non-invasively acquired information about BM and brain phenotypes that could be investigated for their potential to determine before treatment which patient has a high risk of developing ARE. The early identification of these patients is an unmet clinical need which may help in clinical decision making by informing the patients of the risk of ARE, the early risk stratification of patients that may develop ARE, and the consideration of ARE risk mitigating strategies such as deferring radiotherapy for central nervous system-penetrant systemic therapy.

Advanced quantitative medical image analysis methods such as radiomics and deep learning (DL) extract large amounts of imaging features and associate these with biological and/or clinical outcomes using machine learning (ML) techniques (21–26). Thus, radiological images from routine imaging procedures could potentially be used to non-invasively quantify the lesion phenotype, providing clinically necessary information for patient management decisions. Several studies have indicated that MRI radiomics analysis is able to differentiate BM from glioblastoma (27, 28) to predict local recurrence (29, 30), to predict the origin of metastases (31, 32), and to predict overall survival (33, 34). DL has also shown potential in predicting treatment response on brain MRI (35). Moreover, DL and radiomics can have a complementary value, potentially establishing a more robust classifier (36).

We hypothesize that models trained with radiomics features, DL features, and patient characteristics or a combination thereof can predict the occurrence of ARE in patients with BM, both lesion specific and patient specific.

## 2 Materials and methods

### 2.1 Patient Characteristics

All data from patients with BM treated with SRT between 1997 and 2017 for which imaging, outcome data, and patient data were available were collected retrospectively from the University of California—San Francisco (UCSF) medical center’s picture archiving and communication system. Available imaging data, outcome data, and patient data of all patients with BM treated with SRS/SRT between 2014 and 2019 at the University Hospital Zürich (USZ) were collected retrospectively. The data included clinical and biological information for both the patient and the lesion. The eligibility criteria included radical treatment for metastatic brain cancer using Gamma Knife SRS for the UCSF patients and SRS/FSRT for the USZ patients. The inclusion of patients was regardless of the number of BM, but pathohistological or imaging-based confirmation of ARE during the follow-up was required in addition to pathohistological confirmation of the primary tumor. For the USZ cohort, in case of imaging-based suspicion of RN, positron emission tomography imaging was additionally used to exclude TP. The effort obtained ethical approval for observational research using anonymized linked care data for supporting medical purposes that are in the interests of individuals and the wider public. UCSF Institutional Review Board (https://irb.ucsf.edu) and Cantonal Ethics Committee Zurich approval with waiver of informed consent was obtained.

The UCSF dataset was divided randomly into sub-cohorts for training (70%) and testing (30%) while maintaining the ratios of events to non-events equal in both groups. The USZ dataset was used as an independent external validation dataset, *i*.*e*., it was entirely unseen by the models during the training and testing phases. The binary outcome used in training and validation was ARE per lesion, defined as either pathologically or imaging-based confirmation of RN occurring at any time after treatment. For both the USCF and USZ patients, ARE was confirmed by histopathology when treated with open surgery. In all other cases, ARE was confirmed either at routine re-staging 3 months after radiotherapy for asymptomatic patients or at the onset of new symptoms. When patients presented new symptoms, imaging was performed usually after awaiting the effects of cortisone administration. As the time of BM formation is unknown, the outcome was not defined as right-censored. As every lesion is able to independently develop ARE after treatment, every lesion was considered to be an independent sample. The probability of ARE occurring for any lesion within a patient as an outcome was also investigated, whereby each patient was treated as an independent sample instead.

### 2.2 MR Acquisition Parameters and Lesion Segmentation

**Figure 1:**
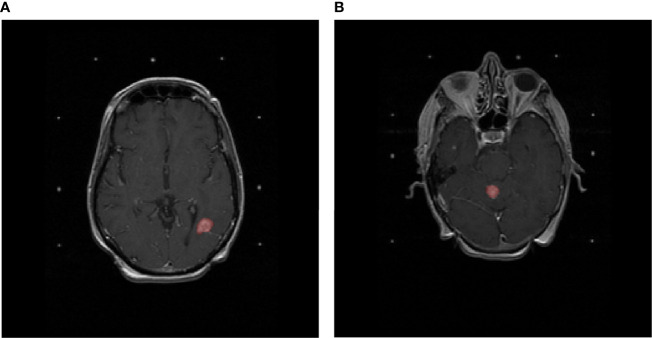
T1-weighted gadolinium-enhanced MRIs of the brain. Delineated in red **(A)** is a lesion that developed adverse radiation effects after stereotactic radiotherapy and **(B)** a lesion that did not develop adverse radiation effects after stereotactic radiotherapy.

All images were axial gadolinium-enhanced T1-weighted MRI acquired prior to the treatment of BM. All included lesions were three-dimensionally delineated for curative Gamma Knife SRS treatment purposes for the UCSF cohort and for curative SRS/FSRT purposes for the USZ cohort according to local protocols by an experienced radiation oncologist. **Figure 1** shows two T1-weighted gadolinium-enhanced MRI with lesions delineated for SRT purposes.

To perform segmentations of the brain and the ventricles on the entire dataset, an atlas-based segmentation strategy was chosen. To create the atlas in the MIM software package (MIM v. 6.9.4, MIM Software Inc., Cleveland, OH, USA), 50 randomly chosen MRI were manually segmented by an expert radiologist.

### 2.3 Pre-Processing of Brain MRI Data

Bias-field correction was performed in the MIM software package using the N4 algorithm, which required brain segmentations (37). A bias field is a low-frequency signal distributed over an MR image, which is caused by inhomogeneities in the magnetic field of the MRI scanner. This causes shifts of intensity value ranges across the image (38). The ventricle mask was subtracted from the brain mask to obtain a white- and gray-matter segmentation. This segmentation was used to determine and correct the bias field present in the image using the N4 algorithm (37) using the MIM software package.

**Figure 2:**
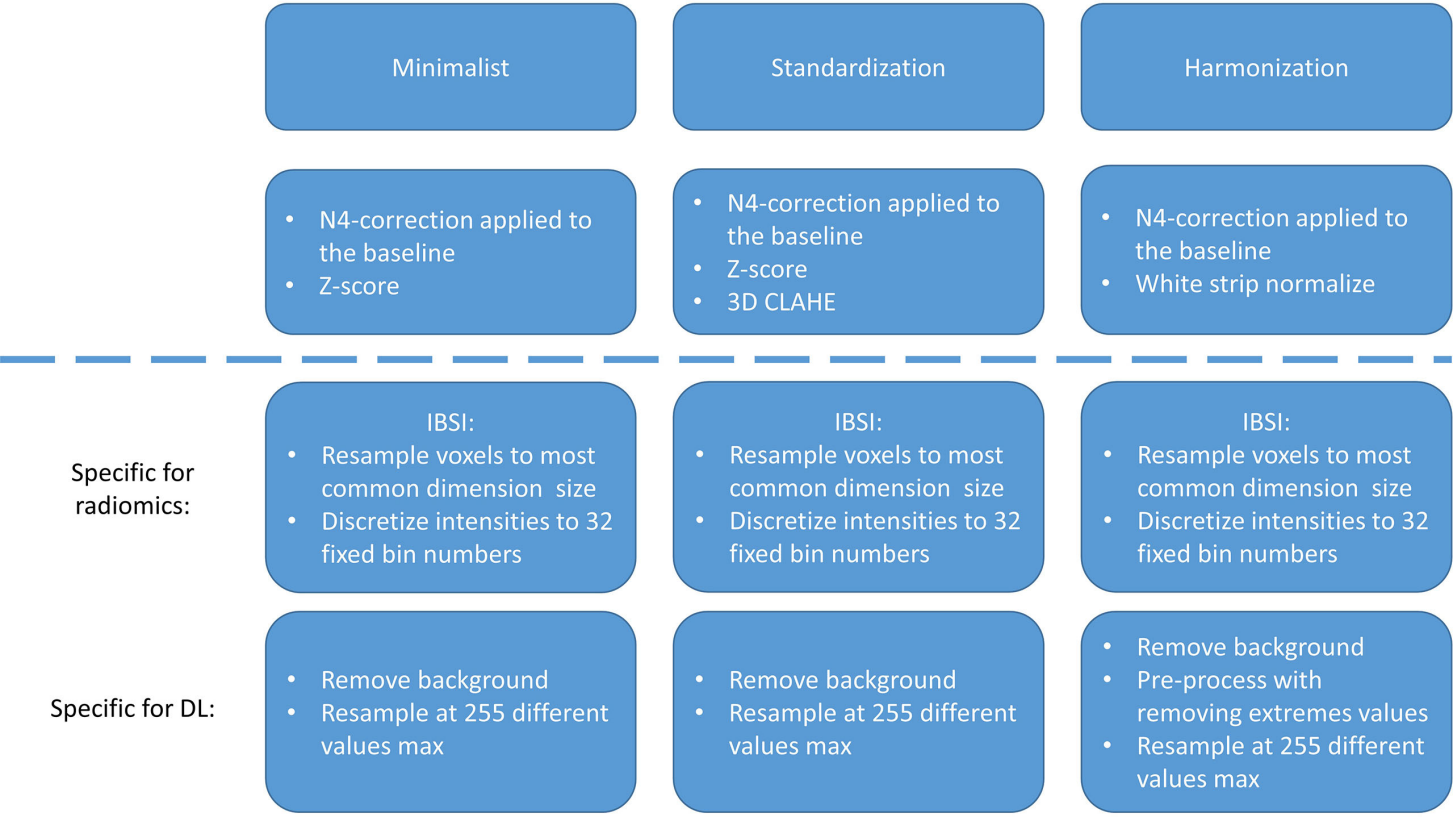
Pre-processing strategies for the “minimalist”, “standardization”, and “harmonization” approaches.

Following the bias correction, all remaining pre-processing, feature extraction, model training, and evaluation were performed in Python (version 3.7). The different Python packages used during this study can be found in **Supplementary Table S1**. Pre-processing of MRI is essential for ML purposes, for reducing scanner dependence, and for ensuring reproducibility (39–41). As there is, to date, no consensus regarding the best way to pre-process MRI for our purposes, three different pre-processing workflows were applied and compared: “minimalist”, standardization, and “harmonization”. The descriptions of these pre-processing workflows can be found in the **Supplementary Materials** (Section 1 and in **Figure 2**).

#### 2.3.1 Pre-processing for radiomics and feature extraction

Feature extraction was performed according to the Image Biomarker Standardization Initiative (IBSI) guidelines (42–44) on the three different sets of processed MRI scans using the BM segmentations. All images were resampled to uniform 1 × 1 × 1-mm**^3^** voxels using the “sitkBSpline” interpolator to correct for differences in pixel size and slice spacing. The choice for voxel dimensions was made based on majority ruling, as it was found that most patients had a pixel spacing of ~1 mm. To achieve isotropic voxels, the choice for resampling in the z-direction was also chosen as 1 mm. Pixel intensity values were resampled to a fixed number of 64 bins, as the number of gray levels was found to affect the interchangeability of MRI radiomics features, and a fixed bin number of 64 has been found recommended in previous studies (42–44).

A total of 106 IBSI features were extracted from each segmentation. The features were extracted from the BM segmentations of the pre-processed images and can be divided into first-order intensity, histogram statistics, shape, and texture features. A full list and a description of the features can be found in the PyRadiomics documentation ([Radiomic Features—PyRadiomics Documentation, (45)], and a description of the feature groups can be found in the **Supplementary Materials** (Section 2).

#### 2.3.2. Pre-processing for deep learning

To inform the DL model on the location and extension of the lesions, lesion masks were used to highlight the ROI. A Gaussian smoothing filter was applied to the image, gradually decreasing the intensity values around the lesion from a factor of 1.0 to 0.2 to still include information of the voxels immediately around the lesion masks.

**Figure 3:**
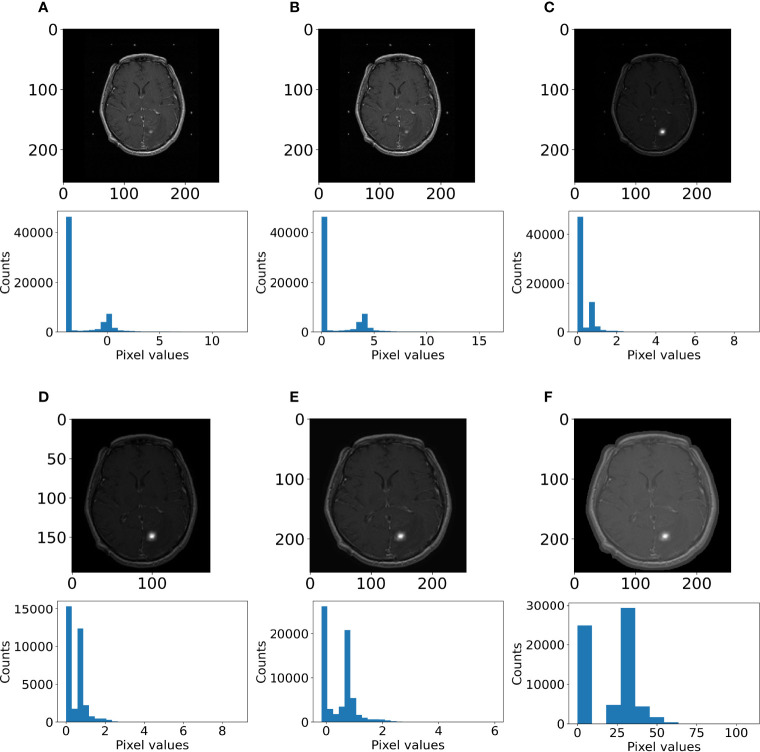
Example of pre-processing strategy: deep learning on the “minimalist” approach. The different steps of preprocessing were **(A)** z-score normalization, **(B)** shift to positive values only, **(C)** pixel attenuations with Gaussian smoothing filtering, **(D)** cropping around the largest bounding box and background set to 0, **(E)** resizing at 256 × 256, and **(F)** rescaling the pixel value range to 0–255.

Otsu thresholding was performed to create a mask containing the brain and the skull. This mask was used to determine the largest three-dimensional bounding box containing the brain and the skull to crop the images. Anything outside this mask was defined as the image background, for which all pixel values were set at 0. For the “minimalist” and the “standardization” datasets, the intensities were resampled in a range between 0 and 255. Finally, the scans were rescaled at 256 × 256 × 64 with spline interpolation order 3. As an example, the steps of the pre-processing workflow for the “minimalist” normalization are illustrated in **Figure 3**.

### 2.4 Machine Learning Models

The mean and SD of each feature over the entire training population were determined. These values were used to apply z-score normalization to the features of the training, testing, and external validation datasets (46). Next, features with low variance (<0.01) were determined and excluded from the dataset. Lastly, the correlation between features was determined using absolute pairwise Spearman rank correlation. As highly correlated features (>0.85) were assumed to contain overlapping information about the outcome, the feature with the highest mean absolute correlation with the rest of the features was excluded. Lastly, supervised feature selection was performed through recursive feature elimination (RFE). RFE uses a ML algorithm to build a multivariate model and determine predictive performance using the currently selected features. It recursively drops and adds features, determining the optimal number of features and the selection of most predictive features.

An extreme gradient boosting (XGBoost) model was used for RFE and ARE prediction. A description of the XGBoost architecture and the methodology to determine the optimal hyperparameters for the trained models can be found in the **supplementary materials** (Section 3).

### 2.5 Deep Learning Model

An Xception three-dimensional model was trained and tested on the same datasets as the handcrafted radiomics-based model. Xception is the extreme version of an Inception model (47), which uses depth-wise separable convolutions. The architecture can be found in **Supplementary Figure S1**. Adam optimization was used (48) with an initial learning rate of 10^-5^, which updated the learning rate during training, and used for loss function binary cross-entropy. This model produced a score ranging from 0 to 1, indicating the estimated probability that a lesion develops ARE. The area under the curve (AUC) of the receiver operating characteristic (ROC) was monitored on the test dataset. The ROC displays the discriminative performance of a model expressed through the sensitivity and specificity as the threshold for binary classification is shifted. The AUC of the ROC is a metric from 0 to 1, where 1 means that the model has perfect predictive performance and 0.5 is equivalent to guessing. To limit the imbalance of the outcomes to affect the model training, the model was only trained on lesions for those patients who had at least a single ARE and tested on the scans of the patients who had ARE in the test dataset. To combine DL and radiomics, the last fully connected layer consisting of 256 features obtained after training the model was extracted. These features were then used to train a ML model similarly to using radiomics features and used in models combining radiomics features and patient characteristics.

### 2.6 Clinical and Treatment-Related Feature Model

As the training and testing datasets contained patient characteristics not available in the external validation dataset, any feature not overlapping between these datasets was dropped. The list of the remaining features was as follows: primary tumor location, primary tumor histology, primary tumor controlled, extra-cranial metastases presence, patient age, patient sex, SRS to the same location, prior external beam radiotherapy (EBRT), prior radiosurgery (RS), neurological symptoms, headaches, seizures, hypertension, diabetes, connective tissue disorder, Karnofsky performance score (KPS) status, prescription dose, and isodose lines. For XGBoost to be able to handle categorical variables, one-hot encoding was performed on two categorical clinical features (primary tumor location and primary tumor histology).

Missing values were imputed using MissForest. MissForest is an imputation algorithm that uses RandomForest to train a model on the non-missing data for each feature with missing values to predict the missing values. In the first iteration, all values are set to the mean value present for each variable (*i*.*e*., each column). Then, over multiple iterations, each data column with missing values will be predicted using all the data except for the rows containing the missing values in question. This process is repeated over several iterations.

### 2.7 Metrics Used for Data Analysis

The patient and tumor characteristics in the UCSF and USZ cohorts were assessed through a two-proportion *z*-test to test for significant differences in categorical variables between the cohorts or the unpaired two-sample *t*-test to test for significant differences in numerical variables. For the latter, the assumptions of the data having a normal distribution and possessing the same variance in both cohorts were tested through Shapiro–Wilk’s test and *f*-test, respectively. The significance level was set at 5%.

To determine which method ensured best performance for the radiomics-based and DL models, models were trained on the three different pre-processed datasets, and the best AUC of the ROC on the testing set was used to determine the best pre-processing methods for ML and DL separately. The 95% confidence intervals (CI) displayed on the ROC curves were obtained using bootstrapping (*n* = 2,000). For the radiomics-based model, the results were reported on the full train dataset and the entire test dataset. For the DL model, the results were reported on the balanced train dataset (which served to train the different DL models) and the full test dataset.

**Figure 4:**
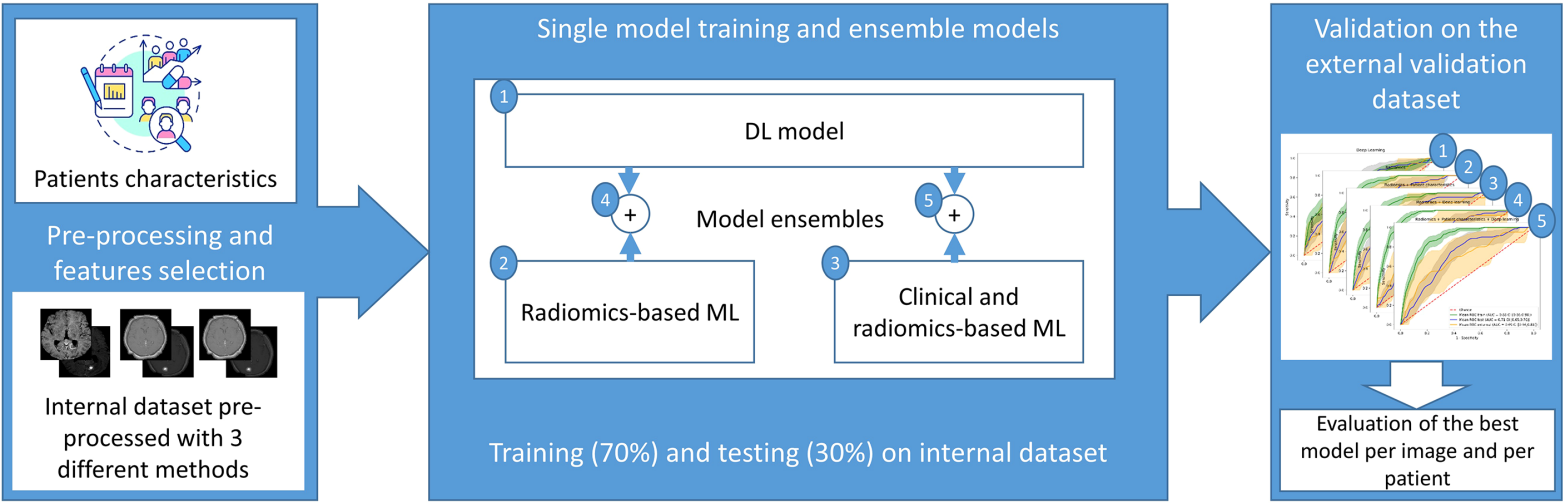
General workflow of the model training process: first, the MRI data was pre-processed using 3 pre-processing methods, the most suitable pre-processed set of images was selected according to the radiomics-based model or the DL model performance on the internal test dataset, then the models were ensembled or trained separately, and finally the performance of each model was computed on the external dataset.

Once the best models were selected, the models were validated on the external dataset. The predictive performance of each model was expressed through the ROC curve and its AUC on the training, testing, and external data. By determining an optimal threshold value using Youden’s J statistic (49) based on the training dataset, a binary classification was performed on the external dataset. From this binary classification, the balanced accuracy, precision, recall, and F1-score were determined. The confusion matrices were also derived from the binary classification. To determine model performance and to compare between models, the recall was investigated specifically, which is the proportion of true positives of the total number of true cases. As the number of events was relatively low and not missing any patients at risk of ARE is crucial, a high recall of the models was desirable. The CI obtained for all metrics were obtained using bootstrapping, resampling the results 2,000 times. Moreover, an analysis of the agreement prediction between the DL model and the radiomics-based model was performed. To give a prediction per patient, the maximum prediction of ARE among the different lesion predictions of the patient was selected. The ground truth to which the prediction was compared with was the ARE status of the patient, meaning that the patient had at least one ARE lesion. An overview of the models tested can be found in **Figure 4**.

We evaluated on the external dataset for which cases the DL model and the best radiomics classifier obtained the same predictions and reported the number of cases for which those models agreed on the label. The metrics based on the data for which the models agreed was also reported.

## 3 Results

### 3.1 Patient Characteristics

A total of 1,404 patients with 7,974 lesions from UCSF and 237 patients with 646 lesions from USZ were included. **Table 1** shows an overview of the patient characteristics of the UCSF and USZ data. Significant differences between the proportion of male and female patients between the datasets (*P* < 0.01), median age (*P* = 0.03), KPS status (*P* < 0.01), and the number of lesions per patient at treatment (*P* < 0.01) were found. Furthermore, the proportions of primary tumor (lung, melanoma, and breast) were different between the datasets, and the data from USZ did not have kidney, GI, sarcoma, or other types of primary locations that were present in the UCSF dataset. For the histology of the primary tumor, only the melanoma histology subtype was found to be present in a significantly different proportion.

**Table 1:** Patient characteristics of University of California—San Francisco (UCSF) and University Hospital Zurich (USZ) datasets.

Patient/tumor characteristic		Total UCSF data	USZ data	*P*
		*N* = 1,404	*N* = 237	
Sex (%)	Male	571 (41)	128 (54)	<0.01
	Female	833 (59)	109 (46)	
Median age ± SD		59 (13)	62 (12)	0.03
KPS (%)	80–100	1,053 (75)	198 (83)	<0.01
	40–80	351 (25)	37 (16)	<0.01
	10–40	0 (0)	2 (1)	–
Primary tumor location (%)	Lung	530 (38)	136 (58)	<0.01
	Breast	357 (25)	27 (11)	<0.01
	Melanoma	272 (19)	74 (31)	<0.01
	Kidney	91 (7)	0 (0)	–
	Gastrointestinal	57 (4)	0 (0)	–
	Gynecologic	27 (2)	0 (0)	–
	Sarcoma	20 (1)	0 (0)	–
	Other	50 (4)	0 (0)	–
Histology primary tumor (%)	Adenocarcinoma	802 (57)	124 (52)	0.17
	Melanoma	272 (19)	74 (31)	<0.01
	Renal cell carcinoma	88 (6)	0 (0)	–
	Small cell carcinoma	44 (3)	0 (0)	–
	Squamous cell carcinoma	40 (3)	10 (4)	0.26
	Sarcoma	18 (1)	0 (0)	–
	Large cell carcinoma	9 (0.6)	2 (1)	0.72
	Bone carcinoma	8 (0.6)	0 (0)	–
	Adeno squamous carcinoma	6 (0.4)	0 (0)	–
	Broncho alveolar cell carcinoma	5 (0.4)	0 (0)	–
	Germ cell carcinoma	2 (0.1)	0 (0)	–
	Lymphoma	1 (0.1)	0 (0)	–
	Other/NOS	109 (8)	27 (11)	0.06
Primary controlled		974 (70)	149 (63)	0.05
ECM present		1,097 (78)	190 (80)	0.48
Number of lesions per patient at treatment	Median ± SD	3 (7)	2 (3)	<0.01
Symptoms	Headaches	437 (31)	31 (13)	<0.01
	Hypertension	407 (29)	0 (0)	< 0.01
	Seizures	134 (10)	16 (7)	0.17
	Diabetes	98 (7)	13 (6)	0.4
	CTD	21 (2)	2 (1)	0.43
Number of lesions in total		7,974	646	–
Number of ARE cases (% of total lesions)		217 (2.7)	20 (3.1)	0.61
Number of patients with ARE (% of total patients)		155 (11)	19 (8)	0.16
Prescription dose ± SD (Gy)		18.5 (1.5)	20 (5.0)	–

The P-value of two-proportion z-test or unpaired two-sample t-test for significant differences between datasets was reported for each characteristic if applicable.

SD, standard deviation; KPS, Karnofsky performance score (80–100, good performance; 50–70, medium performance; and 10–40 bad performance); ECM, extracranial metastasis; BM, brain metastasis; CTD, connective tissue disorder; ARE, adverse radiation effect; Gy, gray.

### 3.2 Radiomics-Based Model and DL Model Results Based on the Three Different Preprocessing Methods of the Dataset

The best AUC on the test dataset for the radiomics-based models was found using the “harmonization” normalization, with an AUC of 0.76 (CI of 0.70–0.81), compared with 0.75 (CI of 0.70–0.80) and 0.73 (CI of 0.67–0.79) for the “minimalist” and “standardization” methods, respectively.

**Figure 5:**
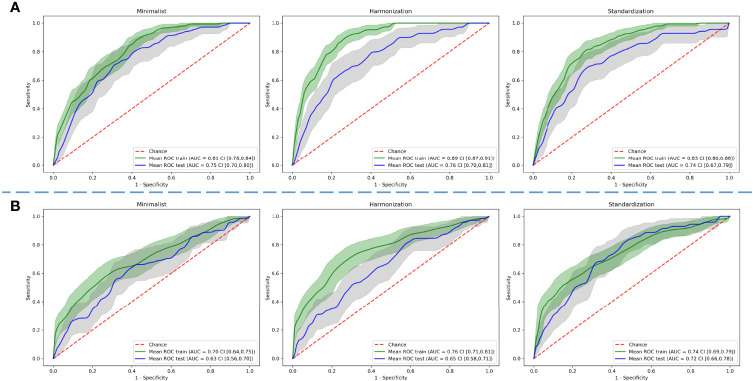
Comparison of predictive performance through receiver operating characteristic curves for **(A)** radiomics-based machine learning and **(B)** deep learning models using three different pre-processed image datasets. The shaded areas represent the 95% confidence intervals of the corresponding receiver operating characteristic curves.

The best AUC on the test dataset for the DL models was found using the “standardization” normalization, with an AUC of 0.72 (CI of 0.66–0.78), compared with 0.63 (CI of 0.57–0.70) and 0.65 (CI of 0.58–0.71) for the “minimalist” and “harmonization” methods, respectively. **Figure 5** shows the ROC curves of the training and testing datasets for the three different pre-processing methods for radiomics-based ML and for DL.

### 3.3 Results of the Combined Best-Performing Models

**Figure 6:**
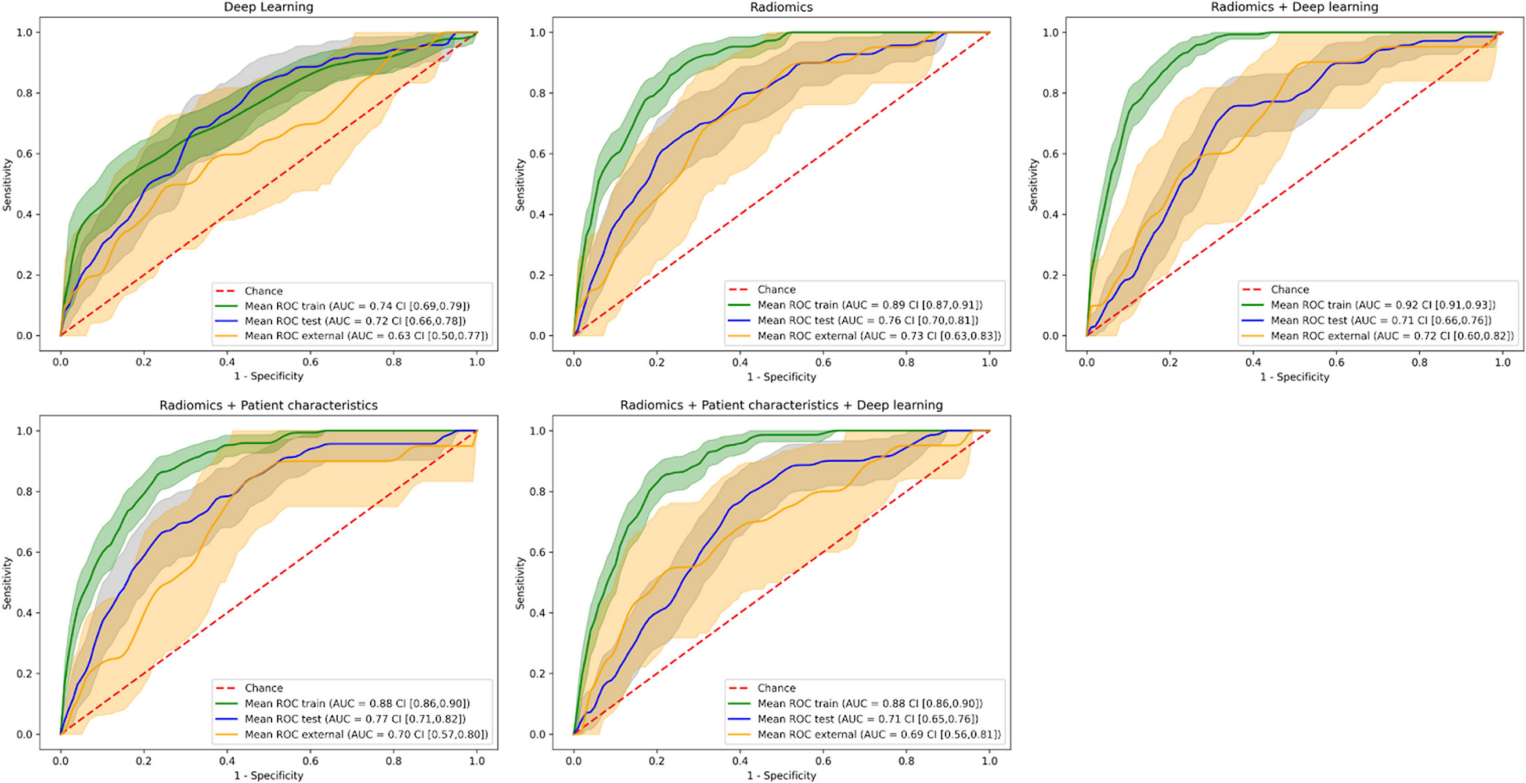
Receiver operating characteristic curves of the training, testing, and external validation datasets for the different model combinations. The shaded areas represent the 95% confidence intervals of the corresponding receiver operating characteristic curves.

We calculated the AUC and CI for each model combination on the external validation dataset. The DL model, built on images pre-processed with the “standardization” method, achieved an AUC of 0.64 (CI of 0.50–0.76). The model built on radiomics features, extracted from the images pre-processed with the “harmonization” method, achieved an AUC of 0.73 (CI of 0.63–0.83). The model was built on 20 features selected through RFE. **Supplementary Figure S2A** provides an overview of the selected features and the corresponding importance in the XGBoost model. **Supplementary Table S2** provides an overview of the hyperparameters determined through grid search cross-validation. The model based on the combination of the DL features extracted from the last layer and radiomics features achieved an AUC of 0.71 (CI of 0.60–0.82). The model was built on 10 features selected through RFE. **Supplementary Figure S2B** provides an overview of the selected features and the corresponding importance in the XGBoost model. The model built on radiomics features, extracted from images pre-processed with the “harmonization” method, combined with patient characteristic features achieved an AUC of 0.70 (CI of 0.57–0.80). The model was built on 19 features selected through RFE. **Supplementary Figure S2C** provides an overview of the selected features and the corresponding importance in the XGBoost model. Finally, the model built on radiomics features, extracted from images pre-processed with the “harmonization” method, combined with DL features, extracted from images pre-processed with the “standardization” method, and patient characteristics achieved an AUC of 0.69 (CI of 0.56–0.81). The model was built on 20 features selected through RFE. **Supplementary Figure S2D** provides an overview of the selected features and the corresponding importance in the XGBoost model. **Figure 6** shows the ROC curves with CI of the training datasets, testing datasets, and validation datasets for these models.

The combination of radiomics and DL features achieved the highest combination of balanced accuracy and recall of 0.67 (CI of 0.56–0.76) and 0.80 (CI of 0.62–0.96), respectively, of the externally validated models for predictions per lesion. For a patient-level prediction, the DL model achieved an AUC of 0.70 (CI of 0.56–0.80) and that of the radiomics model an AUC of 0.72 (CI of 0.60–0.83). A combination of radiomics and DL achieved an AUC 0.71 (CI of 0.57–0.83), that of a combination of radiomics and patient characteristics an AUC of 0.71 (CI of 0.59–0.81), and that of a combination of radiomics features, DL features, and patient characteristics an AUC of 0.72 (CI of 0.58–0.84). The model combining radiomics features, DL features, and patient characteristics achieved the highest combination of balanced accuracy and recall of 0.65 (CI of 0.55–0.74) and 0.84 (CI of 0.65–1.00), respectively, of the externally validated models for predictions per patient. The DL model predictions and the radiomics-based model predictions per lesion agreed for 32% of the external dataset. For the per-patient classification, the DL model predictions and the radiomics combined with clinical feature-based model predictions agreed for 19% of the external dataset. Because the number of patients for which the models agreed was low (47 patients, 6 with ARE), no CI could be derived. **Table 2** provides an overview of the AUC, balanced accuracy, precision, recall, and F1 score metrics for all DL and ML models on both lesion and patient levels and for the agreed labels on the external validation. The corresponding confusion matrices are in **Supplementary Figures S3, S4**, respectively. **Supplementary Tables S3, S4** contain the same metrics as that in **Table 2** for the training and testing datasets, respectively.

**Table 2:** Area under the curve (AUC), balanced accuracy, precision, recall, and F1 metrics with CI on the external validation on patient and lesion levels.

Per-lesion classification						Per-patient classification					
Approaches	AUC	Balanced accuracy	Precision	Recall	F1 score	Approaches	AUC	Balanced accuracy	Precision	Recall	F1 score
Best deep learning model	0.64 CI (0.50, 0.76)	0.57 CI (0.48, 0.64)	0.04 CI (0.02, 0.05)	0.85 CI (0.67, 1.00)	0.07 CI (0.04, 0.10)	Best deep learning model	0.70 CI (0.56, 0.83)	0.63 CI (0.52, 0.73)	0.17 CI (0.09, 0.25)	0.60 CI (0.39, 0.78)	0.26 CI (0.16, 0.37)
Best radiomics model	0.73 CI (0.63, 0.83)	0.62 CI (0.51, 0.74)	0.07 CI (0.03, 0.11)	0.45 CI (0.23, 0.67)	0.12 CI (0.05, 0.19)	Best radiomics model	0.72 CI (0.60, 0.83)	0.59 CI (0.51, 0.69)	0.40 CI (0.09, 0.75)	0.21 CI (0.05, 0.43)	0.28 CI (0.07, 0.48)
Radiomics and DL	0.71 CI (0.60, 0.82)	0.67 CI (0.56, 0.76)	0.05 CI (0.03, 0.08)	0.80 CI (0.62, 0.96)	0.10 CI (0.06, 0.14)	Radiomics and DL	0.71 CI (0.57, 0.83)	0.66 CI (0.54, 0.77)	0.14 CI (0.07, 0.22)	0.63 CI (0.40, 0.84)	0.23 CI (0.13, 0.34)
Radiomics and patient characteristics	0.70 CI (0.57, 0.80)	0.62 CI (0.51, 0.74)	0.06 CI (0.03, 0.10)	0.50 CI (0.28, 0.73)	0.11 CI (0.05, 0.17)	Radiomics and patient characteristics	0.71 CI (0.59, 0.81)	0.57 CI (0.48, 0.68)	0.16 CI (0.04, 0.30)	0.26 CI (0.08, 0.47)	0.20 CI (0.05, 0.35)
Radiomics, DL, and patient characteristics	0.69 CI (0.56, 0.81)	0.64 CI (0.53, 0.74)	0.05 CI (0.03, 0.08)	0.70 CI (0.48, 0.89)	0.09 CI (0.05, 0.14)	Radiomics, DL, and patient characteristics	0.72 CI (0.58, 0.84)	0.65 CI (0.55, 0.74)	0.12 CI (0.07, 0.17)	0.84 CI (0.65, 1.00)	0.21 CI (0.13, 0.29)
Agreed labels	0.67 CI (0.53, 0.81)	0.65 CI (0.53, 0.73)	0.07 CI (0.03, 0.12)	0.90 CI (0.67, 1.00)	0.13 CI (0.06, 0.21)	Agreed labels	NA	NA	NA	NA	NA

## 4 Discussion

Patients with BM treated with SRT are at risk of developing ARE, such as RN. Early identification of these patients can help in clinical decision making. The MRIs required for SRT planning provide an opportunity to identify these patients through quantitative imaging methods. In this large-scale study, ML models that can successfully predict ARE were trained on T1-weighted MR imaging features from secondary brain tumors treated with SRT. As no consensus to harmonize MR images within and between centers exists, multiple methods were tested for the DL and ML pipeline, resulting in two optimal pre-processing methods (“harmonization” for the ML pipeline and “standardization” for the DL pipeline). A ML model trained with radiomics features combined with DL features yielded the highest predictive performance, with a combination of ROC AUC, balanced accuracy, and recall of 0.71, 0.67, and 0.80, respectively. At the patient level, the best-performing ML model was clearly a combination of radiomics, clinical (age at treatment, prior RS, and sex), and DL features achieving the highest predictive performance (AUC of 0.72), a balanced accuracy of 0.65, and recall of 0.84.

Performing an aggregate prediction (*i*.*e*., using only those predictions that agreed on the outcome) did not improve predictive performance for the lesion-level prediction (AUC of 0.67) nor the binary prediction (balanced accuracy of 0.65). However, using this method, the highest recall of 0.90 was achieved, making this method very robust in detecting true positives.

The models pave the way for clinical decision making of patients at risk of ARE before treatment. The information on the risk of an individual patient may be used by clinicians to inform patients of the risk of ARE when SRT is used as treatment. Furthermore, this information may be used to perform an early stratification of those patients at high risk or may allow the patient and clinician to pursue alternative therapy, such as systemic therapy or alternate radiotherapy approaches (*e*.*g*., dose de-intensified SRT or WBRT), if the risk of ARE outweighs the possible benefits of SRT (50).

To our knowledge, this is the first study that performs a pre-treatment prediction of ARE using quantitative image analysis. Several studies have investigated the possibility of differentiating between tumor recurrence and RN after treatment, which is nominally similar in purpose to identify those patients who may have ARE. Zhang et al. (51) used radiomics features extracted from four different MR sequences [T1, T1 post-contrast, T2, and fluid-attenuated inversion recovery (FLAIR)] at two different time-points during follow-up to differentiate RN from TP as confirmed pathologically. A model was built on a dataset of 87 patients with 97 lesions using 5 delta-radiomics features from T1 and T2 sequences. The AUC and binary prediction accuracy of the model were both 0.73. However, this result was obtained using leave-one-out cross-validation, as no external validation was used. Similarly, Peng *et al*. created a model on radiomics features extracted from T1 and T2 FLAIR on 66 patients with 77 lesions in total (52). The model was compared with a neuroradiologist’s performance. No external validation was used, and instead a leave-one-out cross-validation was performed, which gave an AUC of 0.81. The sensitivity and specificity of the neuroradiologist were 0.97 and 0.17, compared with 0.65 and 0.87 for the radiomics-based model. In Park etal. (53), the study compared the results obtained after training radiomics-based models using different MRI sequences [T1, T2, and apparent diffusion coefficient (ADC)]. The models were trained using the data from 86 patients and tested on an external dataset of 41 patients. The best AUC was found on the ADC-based data with 0.80, while the other sequences had AUCs of around 0.65. These results are similar or higher than the results obtained with our model, though within the range of the confidence intervals for the model based on radiomics and DL, and the lack of an external dataset on two of the studies makes the validity of these models difficult to determine (52). Most other studies have a similar lack of external validation and total number of included patients, further making the results difficult to compare with the present study (54). These results show that the model presented in this study is able to perform similarly to or even outperform models that perform classification (post-treatment) instead of prediction (pre-treatment) of ARE.

One of the strengths of the present study is the large number of included patients and subsequent lesions, with 7,974 lesions (2.7% ARE) of 1,404 patients in training and testing and 646 lesions (3.1% ARE) of 237 patients in the external validation. This provides a large volume of data for our models to train on, ensuring that it covers the wide variability found between patients. In addition, the inclusion of an external validation is another strength, especially seeing the general lack of one in most other studies investigating ARE. This ensures that the reported result is not too optimistic and shows that our model can be generalizable to populations from a different hospital in a different country and even with different treatments from the training and testing sets. While the difference in treatment between the training (exclusively SRS) and external validation (a mix of SRS and FSRT) may induce variability due to small differences in treatment planning for these methods, literature has shown that these methods carry the same risk of ARE and were therefore considered interchangeable (16, 17, 19).

The large confidence interval on the external validation is partially due to the low number of positive findings in this dataset (*n* = 20). This is because of the large imbalance in outcomes for both ARE and tumor failure. One of the major problems that may arise from this imbalance is a skewed view of predictive performance. However, this was addressed in the present study through multiple measures. The DL model was trained on a balanced subset of the data that only included patients that suffered at least 1 ARE. For ML, the XGBoost model was trained while scaling the weights of positive and negative classes and the respective proportion of the labels. Finally, through analysis of the confusion matrix, precision recall curves, and recall metric, we ensured that the performance of the model was not entirely driven by labeling the data as the majority class.

While the models have been successfully validated on a dataset from an external center, further validation on multiple centers is required to ensure that the models are generalizable. Future research could therefore focus on validating the present model on other datasets, potentially with recalibration of the model. At a later stage, a clinical trial to test the efficacy of the model is needed to be able to incorporate the model in a clinical setting. A model combining radiomics features, DL features, and patient characteristics with a high accuracy could help choose other treatment options such as surgery only, systemic therapy, or palliative care (55) if the predicted risk of developing ARE is high. The model could also predict if the patient would be at a low risk of developing ARE, in which case SRT could be preferred over other treatment options.

In the present study, only one sequence of the MRI scan was used. Previous studies showed that a combination of radiomics computed on T1 and T2 sequences performs best to differentiate ARE and TP (51, 52), and ADC sequence seems to also show a higher performance (53). Investigating more sequences in a future study may therefore improve the performance of the imaging-based models.

Lastly, for ARE (and, to a lesser degree, TP), treatment is one of the primary factors. In this study, multiple-dose-treatment-related variables have been included, such as prior treatments to the same patients as well as dose variables and the volumes encompassing certain dose levels. However, a more thorough “dosiomics” analysis would probably improve the prediction of ARE. Liang et al. (56) described a method to extract the spatial and texture radiomics features from dose maps (56). They found several radiomics features which have a significant predictive value of radiation pneumonitis. Using a similar method for ARE in BM may result in improved prediction results. Our predictions could also be combined with models automatically classifying tumors and RN on brain MRI, such as in Zhang et al. (51), potentially strengthening the results of those studies.

## 5 Conclusion

Radiomics is able to predict lesions at a high risk of ARE, especially when combined with DL features. When predicting ARE on a patient level, the highest performance was found using a combination of radiomics, DL, clinical, and treatment-related features. These models could potentially be used to aid clinical decision making for patients with BM treated with either gamma knife or EBRT.

## Data Availability Statement

The corresponding author does not own the datasets used (acquired with DTAs). Requests to access the datasets should be directed to olivier.morin@ucsf.edu (for the data from UCSF); Nicolaus.Andratschke@usz.ch (for the data from USZ).

## Ethics Statement

The studies involving human participants were reviewed and approved by the cantonal ethics committee Zurich and University of California San Francisco (UCSF) Institutional Review Board (IRB). Written informed consent from the participants’ legal guardian/next of kin was not required to participate in this study in accordance with the national legislation and the institutional requirements.

## Author Contributions

MB and SK performed all the ML/DL analysis and wrote the manuscript. SK, MV, SB, and OM collected and curated the imaging and patient data from UCSF. SP helped with the ML/DL analysis and study design. HW supervised the progression of the project and the writing of this article and guaranteed the integrity of the analysis and results presented. AC and MV helped with the ML analysis. JT, JK, and NA collected the imaging and patient data from USZ. LH and SB aided with the clinical aspects of the study. PL and OM devised the project’s aim and supervised the progression of the project. All authors contributed to the article and approved the submitted version.

## Funding

The research project has been partially funded by the Clinical Research Priority Program “Artificial Intelligence in Oncological Imaging” of the University of Zurich. PL, HW, MB, SK acknowledge financial support from ERC advanced grant (ERC-ADG-2015 n° 694812 - Hypoximmuno), the European Union’s Horizon 2020 research and innovation programme under grant agreement: MSCA-ITN-PREDICT n° 766276, CHAIMELEON n° 952172, EuCanImage n° 952103 and IMI-OPTIMA n° 101034347.

## Conflict of Interest

LH: none related to the current manuscript, outside of current manuscript: research funding Roche Genentech, Boehringer Ingelheim, AstraZeneca, Takeda (all institution, Beigene under negotiation); advisory board: BMS, Eli Lilly, Roche Genentech, Pfizer, Takeda, MSD, Merck, Novartis, Boehringer Ingelheim, Amgen, Janssen (all institution, Roche one time self); speaker: MSD, Lilly (institution); travel/conference reimbursement: Roche Genentech (self); mentorship program with key opinion leaders: funded by AstraZeneca; fees for educational webinars: Benecke, Medtalks, VJOncology (self), high5oncology (institution); interview sessions funded by Roche Genentech, Bayer, Lilly (institution); local PI of clinical trials: AstraZeneca, Novartis, BMS, MSD, Merck, GSK, Takeda, Blueprint Medicines, Roche Genentech, Janssen Pharmaceuticals, Mirati; PL: none related to the current manuscript; outside of current manuscript: grants/sponsored research agreements from Radiomics SA, Convert Pharmaceuticals and LivingMed Biotech. He received a presenter fee (in cash or in kind) and/or reimbursement of travel costs/consultancy fee (in cash or in kind) from Radiomics SA, BHV, Varian, Elekta, ptTheragnostic/DNAmito, BMS, and Convert pharma. PL has minority shares in the companies Radiomics SA, Convert pharmaceuticals, Comunicare, and LivingMed Biotech, and he is co-inventor of two issued patents with royalties on radiomics (PCT/NL2014/050248 and PCT/NL2014/050728), licensed to Radiomics SA; one issued patent on mtDNA (PCT/EP2014/059089), licensed to ptTheragnostic/DNAmito; one non-issued patent on LSRT (PCT/ P126537PC00), licensed to Varian; three non-patented inventions (softwares) licensed to ptTheragnostic/DNAmito, Radiomics SA and Health Innovation Ventures and two non-issued, non-licensed patents on Deep Learning-Radiomics (N2024482, N2024889). He confirms that none of the above entities or funding sources were involved in the preparation of this paper.

The remaining authors declare that the research was conducted in the absence of any commercial or financial relationships that could be construed as a potential conflict of interest.

## Publisher’s Note

All claims expressed in this article are solely those of the authors and do not necessarily represent those of their affiliated organizations, or those of the publisher, the editors and the reviewers. Any product that may be evaluated in this article, or claim that may be made by its manufacturer, is not guaranteed or endorsed by the publisher.

## Abbreviations

ARE, adverse radiation effects; AUC, area under the curve; AUCPR, area under the precision recall-curve; BM, brain metastasis; CI, confidence interval; CLAHE, contrast-limited adaptive histogram equalization; CTD, connective tissue disorder; DICOM, digital imaging and communications in medicine; DL, deep learning; EBRT, external beam radiotherapy; ECM, extra-cranial metastases; FSRT, fractionated stereotactic radiotherapy; GLCM, gray-level co-occurrence matrix; GLDZM, gray-level distance-zone matrix; GLRLM, gray-level run length matrix; GLSZM, gray-level size-zone matrix; HU, Hounsfield unit; IBSI, Image Biomarker Standardization Initiative; KPS, Karnofsky performance score; ML, machine learning; NGLDM, neighborhood gray-level dependence matrix; NGTDM, neighborhood gray-tone difference matrix; NRRD, nearly raw raster data; RFE, recursive feature elimination; RN, radiation necrosis; ROC, receiver operating characteristic; ROI, region of interest; RS, radiosurgery; SD, standard deviation; SRS, stereotactic radiosurgery; SRT, stereotactic radiotherapy; TP, tumor progression; UCSF, University of California—San Francisco; USZ, University Hospital Zürich; WBRT, whole-brain radiotherapy; XGBoost, extreme gradient boosting.
