# Supplementary material for: Predicting Adverse Radiation Effects in Brain Tumors After Stereotactic Radiotherapy With Deep Learning and Handcrafted Radiomics
Source: Front Oncol. 2022 Jul 13;12:920393. doi: 10.3389/fonc.2022.920393 (PMC9326101; doi:10.3389/fonc.2022.920393)
Supplement: Supplementary file 1 [file Presentation_1.pdf]

Supplementary Material: Materials and methods

1 Pre-processing workflow

Conversion of the data from DICOM to NRRD was done using the "precision-medicine-toolbox" (1) to extract the three-dimensional images. For the first workflow, termed "minimalist", z-score normalization was applied per scan on the white matter only using the "intensity_normalization" package in python 3.7 (2). Z-score normalization refers to the process of normalizing an image by subtracting the mean intensity value from each pixel, and dividing each pixel by the standard deviation (SD) of the intensity histogram. The second pre-processing workflow, termed "standardization", had two steps: z-score normalization as described previously and three-dimensional contrast limited adaptive histogram equalization (CLAHE) applied on the brain using the python package "intensity_normalization", after having rescaled the image intensities to 256 bins. CLAHE applies histogram equalization in small patches of the images to increase image contrast, after which through bilinear interpolation any artificial borders between images are removed (3). The third approach, termed "harmonization", was designed to make the intensities comparable across scans for similar regions of the brain. For this the white stripe normalization which is described in (4), was applied using the python package "intensity_normalization". It has the advantage of harmonizing the images based solely on data contained within the MRI, compared to piecewise linear histogram matching, which requires additional information about the dataset (5). An overview of the applied pre-processing methods can be seen in figure 2.

2 Description of the radiomics features

First order and histogram statistics describe the total distribution of voxel intensities over the MR image. Shape and size features describe the three-dimensional spatial dimensions of the tumor. Texture features describe the relative spatial distribution of intensity values within the tumor derived from 6 different matrices that are defined over the region of interest (ROI): gray-level co-occurrence matrix (GLCM) (6), gray-level run length matrix (GLRLM) (7), gray-level size-zone matrix (GLSZM) (8), gray-level distance-zone matrix (GLDZM) (9), neighborhood gray-level dependence matrix (NGLDM) (10), and neighborhood gray-tone difference matrix (NGTDM) (11).

3 Specifications of the XGBoost model classifying ARE versus no ARE

Gradient boosting creates a classification model built on ensemble decision trees. These decision trees make simple, weak predictions on an outcome. The XGBoost model sums up all the individual decision tree predictions to make a final overall prediction which is a measure ranging from 0 to 1 indicating the

estimated probability that a lesion develops ARE. By additively adding new trees, and calculating what the gain of an added tree is by calculating a loss function of the overall model performance, trees are either selected or pruned.

The XGBoost model contains a number of hyperparameters that regulate training. To prevent the imbalance in outcome from affecting model training, the ratio of true events to controls was used as the weights for positive and negative classes of the model. For feature selection, the following default hyperparameter values were used to define an initial list of predictive features: the maximum depth of a single decision tree (6), the minimum sum of instance weight a node in a decision tree needs to be added (1), the number of decision trees to build the final model (100), the gamma or the minimum loss reduction needed to add a tree (0), and lastly the learning rate (0.3). To find the optimal values for feature selection a grid search with cross-validation (k=10) was performed using these initial features. Grid search is a tuning technique which trains different models, based on all the possible combinations of hyperparameters to test, to determine an optimal set of parameters. It finds this best estimator based on the combination of parameters that produces the highest score, optimizing the area under the precision recall-curve (AUCPR). A 10-fold cross validation grid search was performed for the following XGBoost parameters with corresponding value ranges: the maximum depth of a single decision tree (1-5), the minimum sum of instance weight a node in a decision tree needs to be added (1-6), the number of decision trees to build the final model (10-500 in steps of 10), the gamma or the minimum loss reduction needed to add a tree (0.3-0.5), and lastly the learning rate (10-1, 10-2, and 10-3). Default parameters of the XGBoost model further included the learning task (logistic regression), the weights of positive and negative classes (set to proportion of ARE to non ARE = 0.03), the subsample ratio of columns when constructing each tree (0.7), and the evaluation metric (area under the PR curve). A total of 4800 (10 folds for 480 candidates) folds were fitted for each feature set. The optimized hyperparameters were then used to perform feature selection again, resulting in the optimized list of selected features. These features were subsequently used to perform another grid search with cross-validation, resulting in a final optimal XGBoost model.

References

1. Primakov S, Lavrova E, Salahuddin Z, Woodruff HC, Lambin P. Precision-medicine-toolbox: An open-source python package for facilitation of quantitative medical imaging and radiomics analysis. *arXiv [eessIV]* (2022) http://arxiv.org/abs/2202.13965

2. Reinhold JC, Dewey BE, Carass A, Prince JL. Evaluating the Impact of Intensity Normalization on MR Image Synthesis. (2018) http://arxiv.org/abs/1812.04652

3. Zuiderveld K. Contrast Limited Adaptive Histogram Equalization. *Graphics Gems* (1994)474–485. doi: 10.1016/b978-0-12-336156-1.50061-6

4. Statistical normalization techniques for magnetic resonance imaging. *NeuroImage: Clinical* (2014) 6:9–19. doi: 10.1016/j.nicl.2014.08.008

5. Nyúl LG, Udupa JK, Zhang X. New variants of a method of MRI scale standardization. *IEEE Trans Med Imaging* (2000) 19:143–150. doi: 10.1109/42.836373

6. Haralick RM, Shanmugam K, Dinstein I 'hak. Textural Features for Image Classification. *IEEE Trans Syst Man Cybern* (1973) SMC-3:610–621. doi: 10.1109/TSMC.1973.4309314

7. Galloway MM. Texture analysis using gray level run lengths. *Computer Graphics and Image Processing* (1975) 4:172–179. doi: 10.1016/S0146-664X(75)80008-6

8. Thibault, Fertil, Navarro, Pereira, Cau. Texture indexes and gray level size zone matrix. *Nuclei Classification PRIP*

9. Thibault G, Angulo J, Meyer F. Advanced statistical matrices for texture characterization: Application to DNA chromatin and microtubule network classification. *2011 18th IEEE International Conference on Image Processing* (2011) doi: 10.1109/icip.2011.6116401

10. Sun C, Wee WG. Neighboring gray level dependence matrix for texture classification. *Computer Graphics and Image Processing* (1982) 20:297. doi: 10.1016/0146-664x(82)90093-4

11. Amadasun M, King R. Textural features corresponding to textural properties. *IEEE Trans Syst Man Cybern* (1989) 19:1264–1274. doi: 10.1109/21.44046
