# Supplementary figures and images for: Predicting Adverse Radiation Effects in Brain Tumors After Stereotactic Radiotherapy With Deep Learning and Handcrafted Radiomics
Source: Front Oncol. 2022 Jul 13;12:920393. doi: 10.3389/fonc.2022.920393 (PMC9326101; doi:10.3389/fonc.2022.920393)

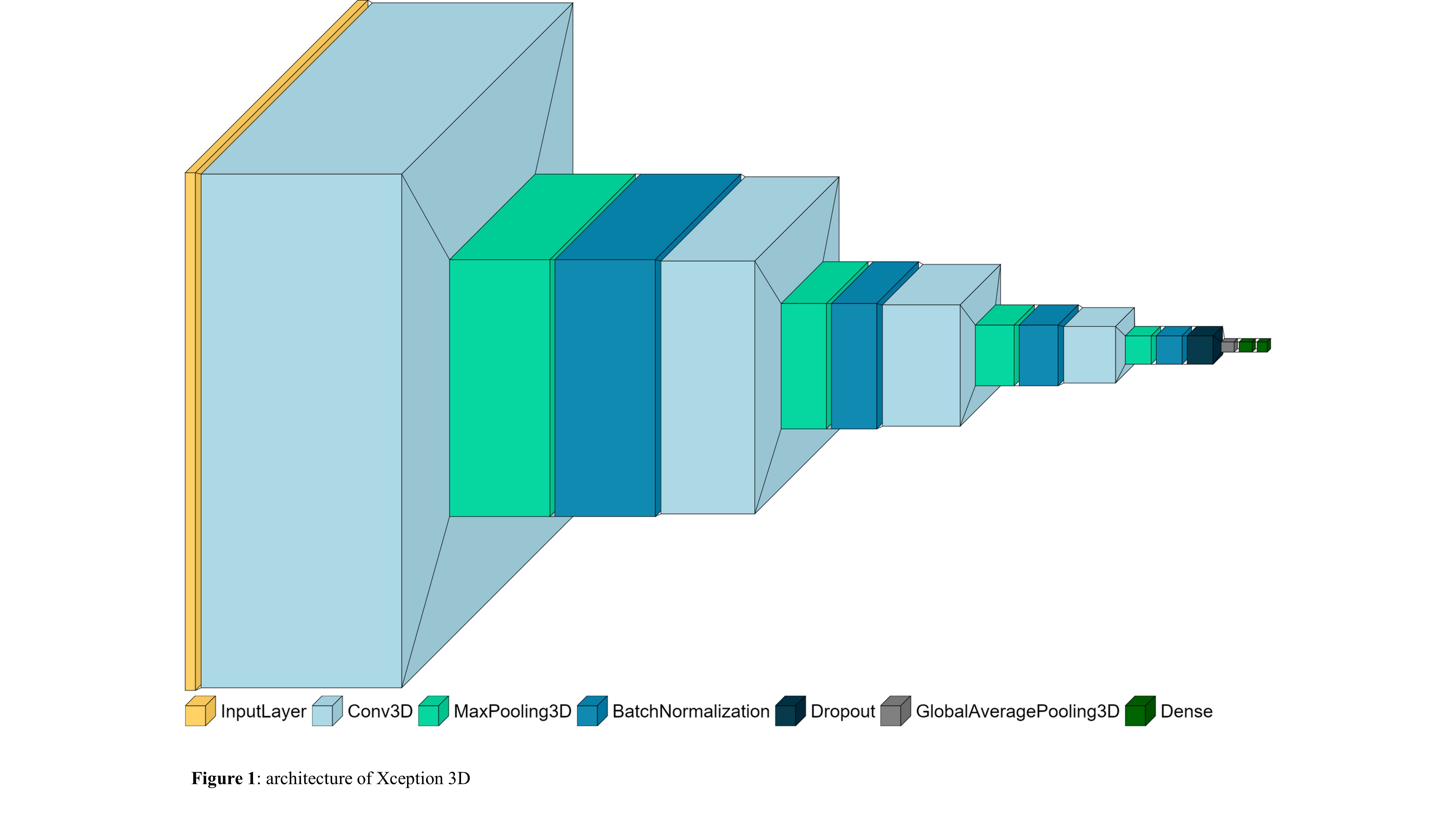

Supplement: Supplementary file 2 [file Image_1.jpeg]

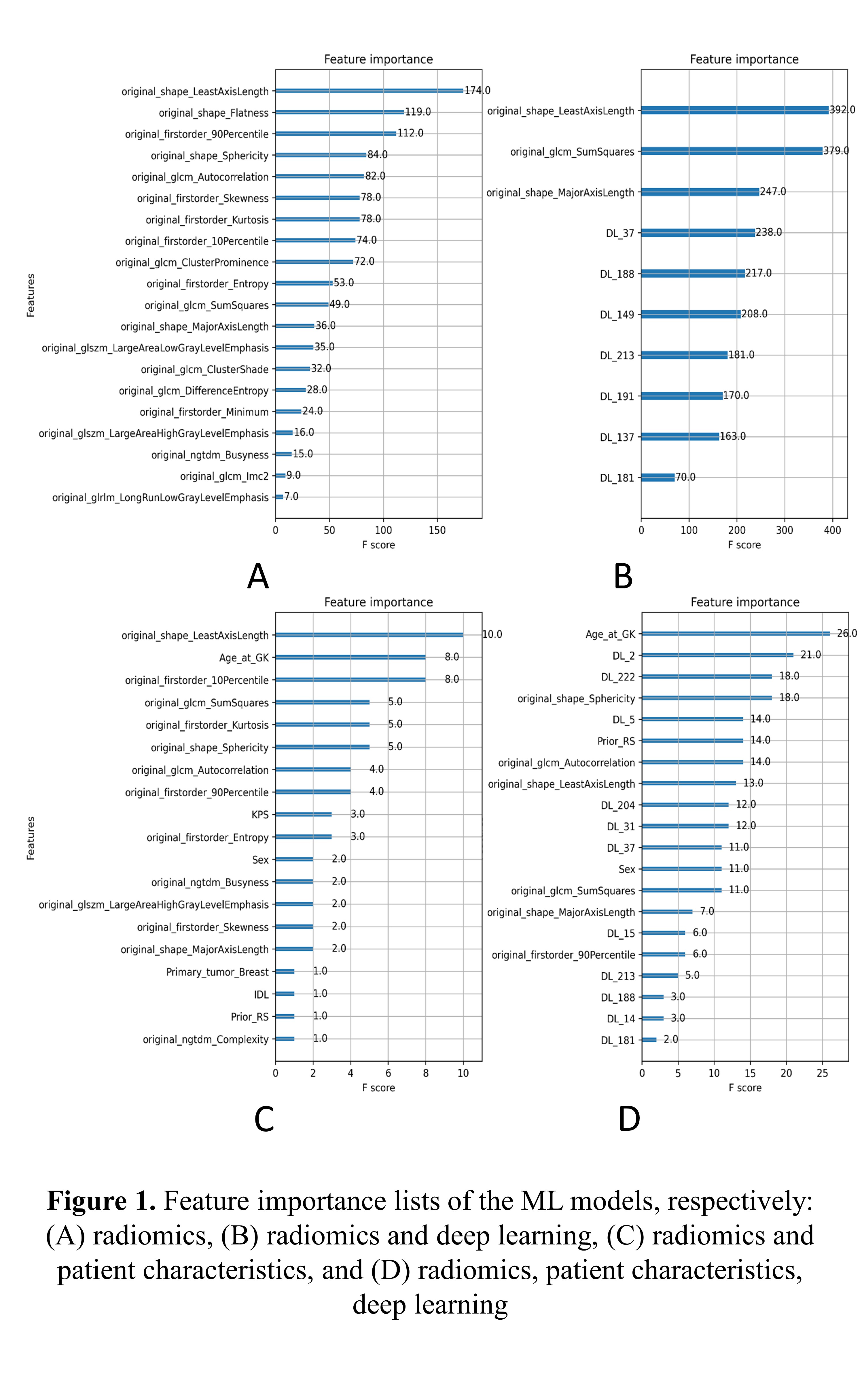

Supplement: Supplementary file 3 [file Image_2.jpeg]

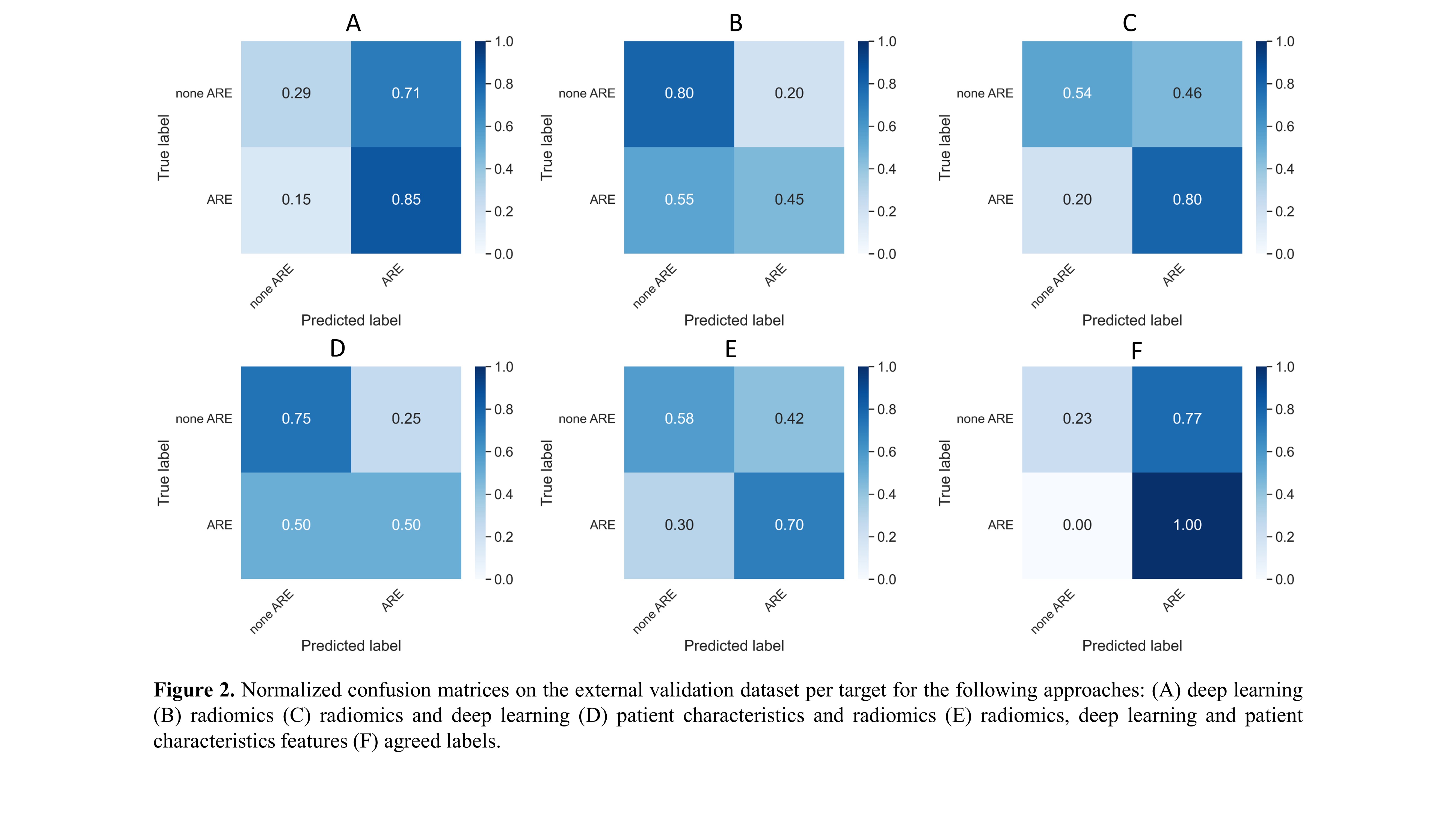

Supplement: Supplementary file 4 [file Image_3.jpeg]

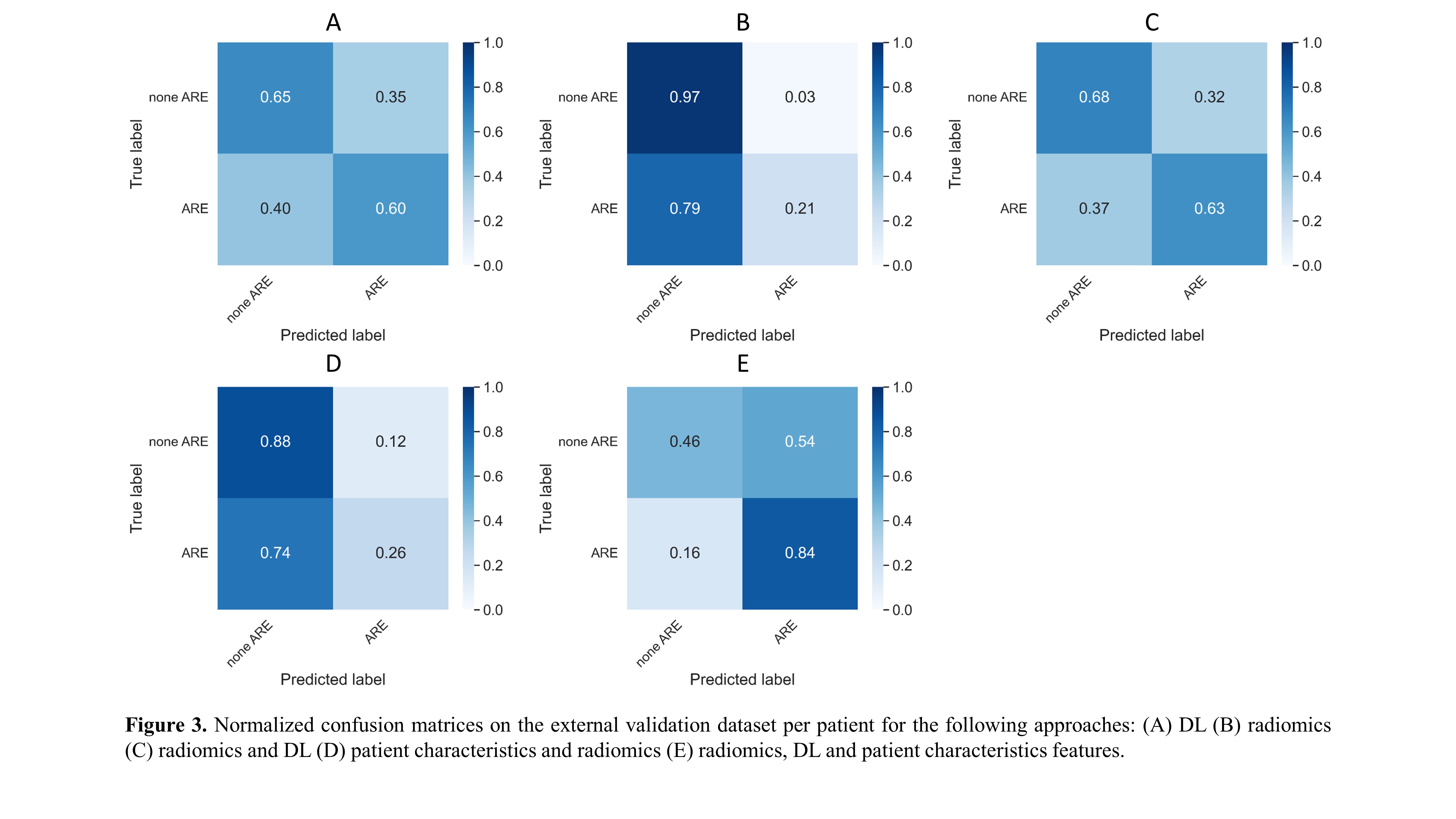

Supplement: Supplementary file 5 [file Image_4.jpeg]
