# Supplementary material for: Predicting Adverse Radiation Effects in Brain Tumors After Stereotactic Radiotherapy With Deep Learning and Handcrafted Radiomics
Source: Front Oncol. 2022 Jul 13;12:920393. doi: 10.3389/fonc.2022.920393 (PMC9326101; doi:10.3389/fonc.2022.920393)
Supplement: Supplementary file 6 [file Table_1.docx]

**Table 1.** Python packages used and their versions.

| purpose | packages | versions |
| --- | --- | --- |
| pre-processing | imutils | 0.5.4 |
|  | intensity-normalization | 2.0.2 |
|  | numpy | 1.19.2 |
|  | opencv | 4.1.0.25 |
|  | os | n/a |
|  | pandas | 0.25.0 |
|  | pydicom | 2.2.2 |
|  | scikit-image | 0.17.2 |
|  | scikit-learn | 0.24.2 |
|  | scipy | 1.5.2 |
|  | simpleITK | 2.1.1 |
| deep learning | keras | 2.3.1 |
|  | tensorflow-gpu | 2.1.0 |
| feature processing and calculation | precision-medicine-toolbox | 0.0.0 |
|  | missingpy | 0.2.0 |
|  | pyradiomics | 3.0.1 |
| machine learning | xgboost | 1.5.1 |
| statistics | statsmodels | 0.13.0 |
| visualisation | matplotlib | 3.3.4 |
