# Supplementary material for: Predicting Adverse Radiation Effects in Brain Tumors After Stereotactic Radiotherapy With Deep Learning and Handcrafted Radiomics
Source: Front Oncol. 2022 Jul 13;12:920393. doi: 10.3389/fonc.2022.920393 (PMC9326101; doi:10.3389/fonc.2022.920393)
Supplement: Supplementary file 7 [file Table_2.docx]

**Table 2.** Overview of hyperparameters optimized through gridsearch cross-validation.

| parameters/data | radiomics only | radiomics + patient characteristics | radiomics + deep learning | radiomics + patient characteristics + deep learning |
| --- | --- | --- | --- | --- |
| gamma | 0,3 | 0,3 | 0,3 | 0,3 |
| learning rate | 0,01 | 0,1 | 0,01 | 0,1 |
| max depth | 3 | 3 | 4 | 1 |
| min child weight | 1 | 1 | 1 | 5 |
| n estimators | 173 | 10 | 173 | 227 |
| number of features selected | 20 | 10 | 20 | 20 |
