# Supplementary material for: Predicting Adverse Radiation Effects in Brain Tumors After Stereotactic Radiotherapy With Deep Learning and Handcrafted Radiomics
Source: Front Oncol. 2022 Jul 13;12:920393. doi: 10.3389/fonc.2022.920393 (PMC9326101; doi:10.3389/fonc.2022.920393)
Supplement: Supplementary file 8 [file Table_3.docx]

**Table 3.** AUC, balanced accuracy, precision, recall, and F1 metrics with CI on the training on patient and lesion levels.

| Per-lesion classification | | | | | | Per-patient classification | | | | | |
| --- | --- | --- | --- | --- | --- | --- | --- | --- | --- | --- | --- |
| **Approaches** | **AUC** | **Balanced accuracy** | **Precision** | **Recall** | **F1 score** | **Approaches** | **AUC** | **Balanced accuracy** | **Precision** | **Recall** | **F1 score** |
| DL | 0.70 [0.66,0.75] | 0.67 [0.63,0.71] | 0.06 [0.05,0.08] | 0.0.56 [0.48,0.64] | 0.11 [0.09,0.14] | DL | 0.58 [0.53,0.64] | 0.58 [0.54,0.62] | 0.11 [0.09,0.13] | 0.73 [0.65,0.81] | 0.19 [0.16,0.23] |
| Rad | 0.89 [0.87,0.91] | 0.81 [0.78,0.84] | 0.09 [0.08,0.11] | 0.86 [0.80,0.01] | 0.17 [0.14,0.19] | Rad | 0.76 [0.72,0.80] | 0.71 [0.67,0.76] | 0.22 [0.18,0.26] | 0.65 [0.55,0.74] | 0.33 [0.27,0.38] |
| Rad + DL | 0.92 [0.91,0.93] | 0.85 [0.83,0.87] | 0.10 [0.09,0.12] | 0.0.93 [0.88,0.96] | 0.18 [0.16,0.21] | Rad + DL | 0.81 [0.78,0.84] | 0.75 [0.71,0.78] | 0.19 [0.15,0.22] | 0.84 [0.77,0.91] | 0.31 [0.26,0.35] |
| Rad + Clin | 0.88 [0.86,0.90] | 0.81 [0.78,0.84] | 0.09 [0.08,0.10] | 0.86 [0.80,0.91] | 0.16 [0.14,0.19] | Rad + Clin | 0.78 [0.73,0.82] | 0.70 [0.66,0.74] | 0.18 [0.14,0.21] | 0.73 [0.64,0.81] | 0.0.29 [0.24,0.33] |
| Rad + DL + Clin | 0.88 [0.86,0.90] | 0.82 [0.79,0.85] | 0.10 [0.08,0.11] | 0.85 [0.79,0.90] | 0.17 [0.15,0.20] | Rad + DL + Clin | 0.77 [0.73,0.81] | 0.70 [0.66,0.73] | 0.15 [0.12,0.18] | 0.88 [0.82,0.94] | 0.25 [0.21,0.29] |
| Agreed labels | 0.88 [0.85,0.90] | 0.82 [0.77,0.85] | 0.09 [0.07,0.11] | 0.81 [0.73,0.88] | 0.16 [0.13,0.19] | Agreed labels | 0.74 [0.69,0.78] | 0.60 [0.58,0.62] | 0.13 [0.11,0.16] | 0.97 [0.93,1.00] | 0.23 [0.19,0.27] |
