# Supplementary material for: Predicting Adverse Radiation Effects in Brain Tumors After Stereotactic Radiotherapy With Deep Learning and Handcrafted Radiomics
Source: Front Oncol. 2022 Jul 13;12:920393. doi: 10.3389/fonc.2022.920393 (PMC9326101; doi:10.3389/fonc.2022.920393)
Supplement: Supplementary file 9 [file Table_4.docx]

| Per-lesion classification | | | | | | Per-patient classification | | | | | |
| --- | --- | --- | --- | --- | --- | --- | --- | --- | --- | --- | --- |
| **Approaches** | **AUC** | **Balanced accuracy** | **Precision** | **Recall** | **F1 score** | **Approaches** | **AUC** | **Balanced accuracy** | **Precision** | **Recall** | **F1 score** |
| DL | 0.72 [0.66,0.78] | 0.61 [0.55,0.67] | 0.07 [0.04,0.09] | 0.37 [0.26,0.49] | 0.11 [0.07,0.16] | DL | 0.63 [0.55,0.71] | 0.59 [0.52,0.66] | 0.12 [0.09,0.17] | 0.63 [0.50,0.77] | 0.21 [0.15,0.27] |
| Rad | 0.76 [0.69,0.81] | 0.70 [0.64,0.76] | 0.07 [0.05,0.09] | 0.67 [0.55,0.78] | 0.13 [0.10,0.16] | Rad | 0.76 [0,70,0.81] | 0.70 [0.65,0.76] | 0.07 [0.05,0.09] | 0.67 [0.56,0.78] | 0.13 [0.10,0.16] |
| Rad + DL | 0.71 [0.66,0.76] | 0.64 [0.58,0.70] | 0.0.06 [0.04,0.08] | 0.0.53 [0.41,0.64] | 0.0.11 [0.08,0.14] | Rad + DL | 0.55 [0.47,0.63] | 0.51 [0.44,0.58] | 0.10 [0.06,0.14] | 0.84 [0.77,0.91] | 0.31 [0.26,0.35] |
| Rad + Clin | 0.77 [0.71,0.82] | 0.71 [0.65,0.76] | 0.07 [0.05,0.09] | 0.69 [0.58,0.79] | 0.13 [0.10,0.16] | Rad + Clin | 0.64 [0.55,0.72] | 0.60 [0.52,0.67] | 0.13 [0.09,0.18] | 0.55 [0.41,0.69] | 022. [0.14,0.28] |
| Rad + DL + Clin | 0.71 [0.65,0.76] | 0.63 [0.57,0.69] | 0.06 [0.04,0.08] | 0.53 [0.41,0.64] | 0.10 [0.07,0.13] | Rad + DL + Clin | 0.59 [0.51,0.67] | 0.65 [0.49,0.63] | 0.11 [0.07,0.15] | 0.65 [0.51,0.78] | 0.19 [0.13,0.25] |
| Agreed labels | 0.81 [0.73,0.89] | 0.73 [0.64,0.81] | 0.10 [0.06,0.15] | 0.55 [0.38,0.71] | 0.17 [0.11,0.24] | Agreed labels | 0.71 [0.62,0.79] | 0.61 [0.59,0.63] | 0.11 [0.08,0.15] | 1.00 [1.00,1.00] | 0.20 [0.14,0.26] |

**Table 4.** AUC, balanced accuracy, precision, recall, and F1 metrics with CI on the internal validation on patient and lesion levels.
